# On the Possibility of Chaos in a Generalized Model of Three Interacting Sectors

**DOI:** 10.3390/e22121388

**Published:** 2020-12-08

**Authors:** Elena V. Nikolova, Nikolay K. Vitanov

**Affiliations:** Institute of Mechanics, Bulgarian Academy of Sciences, Acad. G. Bonchev Str., bl. 4, 1113 Sofia, Bulgaria; elena@imbm.bas.bg

**Keywords:** interacting social sectors, nongovernmental organizations, chaotic attractor, Shilnikov chaos, numerical simulations

## Abstract

In this study we extend a model, proposed by Dendrinos, which describes dynamics of change of influence in a social system containing a public sector and a private sector. The novelty is that we reconfigure the system and consider a system consisting of a public sector, a private sector, and a non-governmental organizations (NGO) sector. The additional sector changes the model’s system of equations with an additional equation, and additional interactions must be taken into account. We show that for selected values of the parameters of the model’s system of equations, chaos of Shilnikov kind can exist. We illustrate the arising of the corresponding chaotic attractor and discuss the obtained results from the point of view of interaction between the three sectors.

## 1. Introduction

Social and economic systems are of high complexity and they are often studied by the methods of nonlinear dynamics and statistical physics [1,2,3,4,5,6]. This allows better understanding of chaos connected to social and economic processes [7,8,9]. One of the possible frameworks for modeling such processes is connected to the ecological determinism [10,11]. Many deterministic ecological models are inspired by the Lotka–Volterra model, which represents the dynamical interactions of two competing species [12,13,14,15,16]. Recently the model has been adapted to describe, for example, dynamics of other subjects, such as interacting populations in presence of adaptation [17,18,19,20,21], financial markets [22,23,24], and socio–economic systems [25,26,27,28]. We note that if the number of competing species in the Lotka–Volterra model becomes three or larger, then chaotic behavior in the phase space can appear [29,30,31].

Below we study a model connected to dynamical interactions among several sectors in a society. In more detail, we extend a version of the generalized Volterra–Lotka model, presented in [11]. This model describes the dynamics of decision-making in the public and private sectors. According to the interpretation of Dendrinos [11], this model can be used to simulate all the possible dynamical interactions between two basic components of decision-making in any social system: for an example, political decision-making and public policy-making including action components and market decision-making elements. Dendrinos wrote a detailed review of modified versions of the generalized Volterra–Lotka model from the point of view of the three broadly defined kinds of economic organization: the Keynesian, Marxist, and Laissez-faire. According to these macroeconomic theories, several variants of the basic model dependent on the types of dynamical ecological interactions between the private and public sectors of a social system (the signs and magnitudes of system parameters) exist. For an example, the Keynesian model corresponds to an ecologically cooperative association between the two sectors, whereas both: Marxist and Laissez-faire models correspond to an isolative association (or predation) between the two economic sectors. In the case of Marxism, the dominant factor is the influence of the state, as the market sector is extinct. The opposite scenario holds for the Laissez-faire model. The general model considered in [11] is

(1) $$\begin{array}{ccc} & & \dot{x}=x\left(a_{10}+a_{11}x+a_{12}y\right)\\ & & \dot{y}=y\left(a_{20}+a_{21}x+a_{22}y\right) \end{array}$$

where x (x>0) is a quantity accounting for the influence of the public decision-making sector, y (y>0) is a quantity accounting for the influence of the private decision-making sector; “dot” presents a time derivative (e.g., $\dot{x}=\frac{dx}{dt}$); and $a_{10},\;a_{11},\;a_{12},\;a_{20},\;a_{21},\;a_{22}$ are interaction parameters whose values can be between −∞ and +∞. The variables *x* and *y* could be any macrovariables depicting the influence of either the public or private sector respectively. Such variables can be, for example, the amounts of investments in the public and private sectors.

Below we shall consider an extension of the model of Dendrinos, keeping in the mind that this is a model for dynamical interactions of decision-making components in a social system. We will differentiate a non-governmental (NGO) sector in addition to the rest of the system which is assumed to be separated into public and private sector. Thus we consider a three-component system, and we are going to study some consequences of the existence of the third component which actively participates in the decision-making at least in some countries where the NGO sector is strong enough.

The basis for our study is the following particular case of (1):

(2) $$\begin{array}{ccc} & & \dot{x}=x\left(a_{10}-a_{11}x-a_{12}y\right)\\ & & \dot{y}=y\left(-a_{20}+a_{21}x+a_{22}y\right) \end{array}$$

The values of the parameters $a_{10},\;a_{11},\;a_{12},\;a_{20},\;a_{21},\;a_{22}$ will be assumed to be nonnegative. The equation for *x* describes logistic growth of *x*, and the increase of *y* leads to a decrease of $\dot{x}$ which is the growth of *x* per unit time (the increase of the influence of the private sector slows the growth of the influence per unit time of the public sector). In addition, the increase of the influence of the public sector leads also to an increase of the growth per unit time of the influence of the private sector. The increase of the influence of the private sector leads to a further increase of the growth per unit time of the influence of the private sector. Thus (2) describes a situation which is favorable for the private sector.

Below we extend the system (2) by adding an ordinary differential equation which describes dynamics of the NGO sector. In more detail, we shall model dynamical interactions among the influences of the public, the private, and the NGO sectors. A short description of the extended model is presented in Section 2 of the paper. Our goal is to demonstrate that chaos based on the Shilnikov theorem can arise in the extended model (we note that chaos is not possible either in the system (1) or in the system (2) and this is a consequence of the Poincare–Bendrixon theorem for a plane). In Section 3 conditions for existence of Shilnikov chaos in the extended model are analytically determined. 3D phase portraits, which illustrate the evolution of a system chaotic attractor, are presented in Section 4. Several concluding remarks are summarized in Section 5.

## 2. Mathematical Formulation of the Problem

Here we shall extend the Dendrinos’s model by adding a new ordinary differential equation to the system (2). The extended model is:

(3) $$\begin{array}{ccc} & & \dot{x}=x\left(a_{10}-a_{11}x-a_{12}y-a_{13}z-a_{14}yz\right)\\ & & \dot{y}=y\left(-a_{20}+a_{21}x+a_{22}y-a_{23}z-a_{24}xz\right)\\ & & \dot{z}=z\left(a_{30}-a_{31}x-a_{32}y-a_{33}z-a_{34}xy\right) \end{array}$$

Above x (x>0) is a quantity accounting for the influence of the public sector; y (y>0) is the quantity accounting for the influence of the private sector; and z (z>0) is the quantity accounting for the influence of the NGO sector. All coefficients in the system (3) are nonnegative. In the model (3) we account for the two-sector interactions and for the interaction between the three sectors (the terms proportional to xyz). The time derivative of a quantity corresponds to the growth of the influence of the corresponding sector per unit time. We assume that an increase of the influence of the NGO sector leads to a decrease of the growth of the influence per unit time of the public sector, and the increase of the product of influences of the private sector and the NGO sector also leads to a decrease of the growth of the influence per unit time of the public sector (this is accounted for by the last term in the first equation of (3). The increase in the influence of the NGO sector is assumed to decrease the growth of the influence per unit time of the private sector and the increase in the product of influence of the public and NGO sectors leads to a decrease of the growth of the influence per unit time of the private sector. In addition, the increase of the influence of the public and private sectors leads to a decrease of the growth of the influence per unit time of the NGO sector; and an increase of the product of the influences of the public and private sectors also leads to a decrease of the growth of the influence per unit time of the NGO sector. Finally, if no interaction between the sectors exists, then the growth of the influence per unit time of the public and NGO sector decreases with increasing influence of these sectors, whereas the growth of the influence per unit time of the private sector increases with increasing influence of the private sector.

The model (3) favors the private sector, and there is competition between the private sector and the NGO sector for influence. The presence of private and NGO sectors decreases the growth of the influence of the public sector and the presence of public and private sectors decreases the growth of the influence of the NGO sector. In the absence of a private sector, the increase of the influence of public and NGO sectors is assumed to follow a logistic law.

The inclusion of third sector in the model opens the possibility for the existence of chaotic motion in the phase space of the quantities *x*, *y*, and *z*. What is interesting is that in the studied case this chaotic motion can be of Shilnikov kind. In more detail, chaotic motion in the model system (3) can exist if the conditions of the theorem of Shilnikov [32] are satisfied. The theorem of Shilnikov is as follows.

**Theorem** **1.** *If for the system*

(4) $$\begin{array}{ccc} & & \dot{x}=\rho x-\omega y+P\left(x,y,z\right)\\ & & \dot{y}=\omega x+\rho y+Q\left(x,y,z\right)\\ & & \dot{z}=\gamma z+R\left(x,y,z\right) \end{array}$$

*where (P,Q,R are $C^{r}$ functions (1<r<∞) vanishing together with their first derivative at O=(0,0,0)), an unstable orbit Γ exists, which is a homoclinic connection, and if*

(5) γ>−ρ>0

*then every neighborhood of the orbit *Γ* contains a denumerable set of unstable periodic solutions of saddle type.*

## 3. Appearance of Shilnikov Chaos in the System (3)

In order to obtain chaotic behavior for the system (3), according to the requirements of the theorem of Shilnikov we have to analyze the properties of the equilibrium points of the system (3). In analogy with [17,18,19,20,21,33] we find out that one of the possibilities for arising of Shilnikov chaos in the system (3) is in the case where the system parameters are described by the following relationships:

(6) $$\begin{array}{c} a_{11}=a_{12}=a_{21}=\eta_{1},\;a_{22}=a_{33}=a_{13}=a_{23}=a_{32}=\eta_{2},\\ a_{31}=\eta_{3},\;a_{14}=a_{24}=a_{34}=\eta_{4} \end{array}$$

and

(7) $$a_{10}=2\eta_{1}+\eta_{2},\;a_{20}=\eta_{1},\;a_{30}=\eta_{3}+2\eta_{2}$$

In this case the number of system parameters is reduced to four: $\eta_{i}\;\left(i=1,2,3,4\right)$, and the system (3) becomes:

(8) $$\begin{array}{ccc} & & \dot{x}=x\left(2\eta_{1}+\eta_{2}-\eta_{1}x-\eta_{1}y-\eta_{2}z-\eta_{4}yz\right)\\ & & \dot{y}=y\left(-\eta_{1}+\eta_{1}x+\eta_{2}y-\eta_{2}z-\eta_{4}xz\right)\\ & & \dot{z}=z\left(\eta_{3}+2\eta_{2}-\eta_{3}x-\eta_{2}y-\eta_{2}z-\eta_{4}xy\right) \end{array}$$

The equilibrium points of (8) are:

(9) $$\begin{array}{ccc} & & E_{1}:\;x=y=z=0,\;E_{2}:\;x=z=0,\;y=\frac{\eta_{1}}{\eta_{2}},\;E_{3}:\;x=\frac{2\eta_{1}+\eta_{2}}{\eta_{1}},\;y=z=0,\\ & & E_{4}:\;x=y=0,\;z=\frac{2\eta_{2}+\eta_{3}}{\eta_{2}},\;E_{5}:\;x=\frac{2\eta_{1}\eta_{2}-\eta_{1}^{2}+\eta_{2}^{2}}{\eta_{1}\left(\eta_{1}-\eta_{2}\right)},\;y=\frac{\eta_{1}+\eta_{2}}{\eta_{1}-\eta_{2}},\;z=0,\\ & & E_{6}:\;x=0,\;y=\frac{1}{2}\frac{2\eta_{2}+\eta_{3}+\eta_{1}}{\eta_{2}},\;z=\frac{1}{2}\frac{2\eta_{2}-\eta_{3}+\eta_{1}}{\eta_{2}},\\ & & E_{7}:\;x=\frac{\eta_{2}+\eta_{3}-2\eta_{1}}{\eta_{3}-\eta_{1}},\;y=0,\;z=\frac{\eta_{1}\eta_{3}+\eta_{2}\eta_{3}-2\eta_{1}\eta_{2}}{\eta_{2}\left(\eta_{3}-\eta_{1}\right)}\\ & & E_{8,9,10,11,12}:\\ & & y=\frac{\eta_{3}\eta_{4}x^{2}-\left(\eta_{1}\eta_{2}+\eta_{2}\eta_{3}+2\eta_{2}\eta_{4}+\eta_{3}\eta_{4}\right)x+\eta_{1}\eta_{2}+\eta_{2}\eta_{3}+2\eta_{2}^{2}}{2\eta_{2}^{2}-\eta_{4}^{2}x^{2}}\\ & & z=\frac{\eta_{1}\eta_{4}x^{2}-\left(\eta_{2}\eta_{3}+\eta_{1}\eta_{2}-\eta_{1}\eta_{4}\right)x+2\eta_{2}^{2}+\eta_{2}\eta_{3}-\eta_{2}\eta_{1}}{2\eta_{2}^{2}-\eta_{4}^{2}x^{2}} \end{array}$$

where $x_{8,\ldots ,12}$ are roots of the equation:

$$\begin{array}{ccc} & & x^{5}-\frac{\eta_{2}\eta_{4}+2\eta_{1}\eta_{4}+\eta_{1}\eta_{2}}{\eta_{1}\eta_{4}}\;x^{4}+\frac{\eta_{1}\left(\eta_{2}\eta_{3}-5\eta_{2}^{2}+\eta_{2}\eta_{4}-\eta_{3}\eta_{4}\right)-\eta_{2}\eta 3\left(\eta_{3}-\eta_{2}\right)}{\eta_{1}\eta_{4}^{2}}\;x^{3}\\ & & +\frac{1}{\eta_{1}\eta_{4}^{3}}(2\eta_{2}^{2}\eta_{3}\eta_{1}-\eta_{1}^{2}\eta_{2}^{2}+7\eta_{4}\eta_{2}^{2}\eta_{1}+\eta_{4}\eta_{1}^{2}\eta_{2}+\eta_{2}^{2}\eta_{3}^{2}+2\eta_{4}^{2}\eta_{2}\eta_{1}+2\eta_{4}\eta_{2}^{3}\\ & & +2\eta_{2}^{3}\eta_{1}+\eta_{4}^{2}\eta_{3}\eta_{1}-\eta_{4}\eta_{2}\eta_{3}\eta_{1}+3\eta_{4}\eta_{2}^{2}\eta_{3}+2\eta_{4}\eta_{2}\eta_{3}^{2})\;x^{2}+\frac{1}{\eta_{1}\eta_{4}^{4}}(-2\eta_{2}^{4}\eta_{3}\\ & & -\eta_{4}^{2}\eta_{1}^{2}\eta_{2}-4\eta_{4}^{2}\eta_{2}^{3}-2\eta_{1}^{2}\eta_{2}^{3}+6\eta_{1}\eta_{2}^{4}-6\eta_{4}\eta_{2}^{3}\eta_{1}-2\eta_{1}\eta_{2}^{3}\eta_{3}-2\eta_{4}\eta_{2}^{2}\eta_{3}^{2}-4\eta_{4}\eta_{2}^{3}\eta_{3}\\ & & +2\eta_{4}\eta_{1}^{2}\eta_{2}^{2}-2\eta_{4}\eta_{2}^{2}\eta_{3}\eta_{1}-4\eta_{4}^{2}\eta_{2}^{2}\eta_{3}-\eta_{4}^{2}\eta_{2}\eta_{3}^{2})\;x+\frac{1}{\eta_{1}\eta_{4}^{4}}(2\eta_{1}^{2}\eta_{2}^{3}+2\eta_{2}^{4}\eta_{3}\\ & & +2\eta_{1}\eta_{2}^{3}\eta_{3}+\eta_{4}\eta_{2}^{2}\eta_{3}^{2}+4\eta_{4}\eta_{2}^{3}\eta_{3}-\eta_{4}\eta_{1}^{2}\eta_{2}^{2}+4\eta_{4}\eta_{2}^{4}-6\eta_{1}\eta_{2}^{4})=0 \end{array}$$

The equilibrium states (9) are realistic (nonnegative) when

(10) $$\eta_{3}>\eta_{1}>\eta_{2}>0,\;\eta_{4}>0$$

According to requirements of the theorem of Shilnikov, chaotic motion for the system (8) will be observed if two appropriate fixed points with different dynamical properties exist [34]. The first of these points must become unstable by means of a local Hopf bifurcation, and the second one must be of a saddle—focus type. The linear stability of the equilibrium points is determined by the Jacobian matrix:

(11) $$M_{ij}=\left(\begin{array}{c} M_{11}-\lambda \;-\eta_{1}x\;-\eta_{2}x\\ \;\eta_{1}y\;M_{22}-\lambda \;-\eta_{2}y\\ \;-\eta_{3}z\;-\eta_{2}z\;M_{33}-\lambda \end{array}\right)$$

where

(12) $$\begin{array}{ccc} & & M_{11}=2\eta_{1}+\eta_{2}-2\eta_{1}x-\eta_{1}y-\eta_{2}z-\eta_{4}yz\\ & & M_{22}=-\eta_{1}+\eta_{1}x+2\eta_{2}y-\eta_{2}z-\eta_{4}xz\\ & & M_{33}=2\eta_{2}-\eta_{3}x-\eta_{2}y-2\eta_{2}z-\eta_{4}xy \end{array}$$

In order to obtain the Hopf bifurcation in our three-dimensional system, we use the center- manifold theorem. In accordance with this theorem we must reduce the considered system to a normal form in which the bifurcation occurs when the system parameters approach 0, and an equilibrium point is located in the origin with pure imaginary characteristic eigenvalues. In our case this equilibrium point is $E_{8}=\left(1,1,\frac{\eta_{2}}{\eta_{2}+\eta_{4}}\right)$ and its eigenvalues are determined by the following characteristic equation:

(13) $$\begin{array}{ccc} & & \left(\eta_{2}^{3}+3\eta_{4}\eta_{2}^{2}+3\eta_{2}\eta_{4}^{2}+\eta_{4}^{3}\right)\lambda^{3}+(\eta_{1}\eta_{4}^{3}+\eta_{4}^{3}\eta_{2}+3\eta_{1}\eta_{2}\eta_{4}^{2}\\ & & -\eta_{2}^{2}\eta_{4}^{2}-\eta_{4}\eta_{2}^{3}+3\eta_{1}\eta_{4}\eta_{2}^{2}+\eta_{4}^{4}+\eta_{1}\eta_{2}^{3})\lambda^{2}+(\eta_{1}\eta_{4}^{4}+\eta_{1}^{2}\eta_{4}^{3}\\ & & -3\eta_{4}^{2}\eta_{2}^{2}\eta_{3}-\eta_{4}^{2}\eta_{2}^{2}\eta_{1}-7\eta_{4}\eta_{2}^{4}-3\eta_{4}\eta_{2}^{3}\eta_{3}+3\eta_{4}\eta_{1}^{2}\eta_{2}^{2}+3\eta_{4}^{2}\eta_{1}^{2}\eta_{2}\\ & & +\eta_{4}^{3}\eta_{2}\eta_{1}-2\eta_{2}^{5}-\eta_{2}^{4}\eta_{3}-3\eta_{2}\eta_{4}^{4}-\eta_{3}\eta_{4}^{3}\eta_{2}-10\eta_{2}^{2}\eta_{4}^{3}+\eta_{1}^{2}\eta_{2}^{3}\\ & & -\eta_{4}\eta_{2}^{3}\eta_{1}-12\eta_{4}^{2}\eta_{2}^{3})\lambda +3\eta_{4}\eta_{2}^{3}\eta_{3}\eta_{1}+\eta_{2}^{5}\eta_{3}+4\eta_{4}\eta_{2}^{4}\eta_{3}-7\eta_{4}^{3}\eta_{2}^{2}\eta_{1}\\ & & +2\eta_{1}^{2}\eta_{2}^{3}\eta_{4}+3\eta_{4}^{2}\eta_{2}^{2}\eta_{3}\eta_{1}+2\eta_{1}^{2}\eta_{2}^{2}\eta_{4}^{2}+2\eta_{1}^{2}\eta_{2}\eta_{4}^{3}+8\eta_{4}^{3}\eta_{2}^{3}\\ & & -11\eta_{4}^{2}\eta_{2}^{3}\eta_{1}-2\eta_{4}^{4}\eta_{2}\eta_{1}+2\eta_{4}\eta_{2}^{5}+\eta_{1}^{2}\eta_{4}^{4}+6\eta_{4}^{2}\eta_{2}^{4}+\eta_{2}\eta_{4}^{3}\eta_{3}\eta_{1}\\ & & +\eta_{1}\eta_{2}^{4}\eta_{3}-3\eta_{2}^{5}\eta_{1}+\eta_{1}^{2}\eta_{2}^{4}+2\eta_{3}\eta_{4}^{3}\eta_{2}^{2}-9\eta_{4}\eta_{2}^{4}\eta_{1}+5\eta_{4}^{2}\eta_{2}^{3}\eta_{3}+2\eta_{2}^{2}\eta_{4}^{4}=0 \end{array}$$

The limit cycle arising by the Hopf bifurcation must increase its size with appropriate change of the system parameters and must come close to the second equilibrium point, which is of a saddle focus type. Then, the Shilnikov theorem states that if a homoclinic orbit forms for the saddle focus, and if $\lambda_{3}>-\rho$, then chaotic behavior will be observed in a parameter range around the value at which the homoclinic orbit arises. For the case studied in this text, the saddle focus is the equilibrium point: $E_{5}=\left(\frac{2\eta_{1}\eta_{2}-\eta_{1}^{2}+\eta_{2}^{2}}{\eta_{1}\left(\eta_{1}-\eta_{2}\right)},\frac{\eta_{1}+\eta_{2}}{\eta_{1}-\eta_{2}},0\right)$. The eigenvalues connected to linear stability of this equilibrium point are:

(14) $$\begin{array}{ccc} & & \lambda_{1,2}=\frac{3\eta_{1}\eta_{2}-\eta_{1}^{2}+2\eta_{2}^{2}\pm \sqrt{13\eta_{1}^{2}\eta_{2}^{2}+2\eta_{1}^{3}\eta_{2}+4\eta_{2}^{3}\eta_{1}-3\eta_{1}^{4}}}{2(\eta_{1}-\eta_{2})},\\ & & \lambda_{3}=\frac{3\eta_{1}\eta_{2}^{3}-4\eta_{1}^{2}\eta_{2}^{2}+\eta_{1}^{3}\eta_{2}+\eta_{1}^{2}\eta_{3}\eta_{2}-\eta_{3}\eta_{2}^{3}+\eta_{4}\eta_{1}^{2}\eta_{2}+3\eta_{4}\eta_{1}\eta_{2}^{2}-\eta_{4}\eta_{1}^{3}+\eta_{4}\eta_{2}^{3}}{\eta_{1}{\left(\eta_{1}-\eta_{2}\right)}^{2}} \end{array}$$

Then, the area of validity of the Shilnikov theorem for the system (8) is determined by the following proposition.

**Proposition** **1.** *When $0<\eta_{2}/\eta_{1}<\frac{\sqrt{17}-3}{4},\;\eta_{3}/\eta_{1}>1,\;0<\eta_{4}/\eta_{1}<\frac{7\sqrt{17}+33}{3\sqrt{17}-5}\beta +\frac{61\sqrt{17}-251}{3\sqrt{17}-5}$, chaotic motion of the Shilnikov kind for the system (8) exists.*

**Proof.**  We denote $\alpha =\frac{\eta_{2}}{\eta_{1}}$, $\beta =\frac{\eta_{3}}{\eta_{1}}$ and $\delta =\frac{\eta_{4}}{\eta_{1}}$, assuming α<1, β>1 and δ>0 to satisfy the condition (10) for the system parameter region. Rewriting Equation (14) in terms of α, β, and δ, we search for a solution of the system of inequalities

(15) $$\begin{array}{ccc} & & \frac{13\alpha^{2}+4\alpha^{3}+2\alpha -3}{2(\alpha -1)}<0,\;\frac{-3\alpha -2\alpha^{2}+1}{2(\alpha -1)}<0,\\ & & \frac{3\alpha^{3}-4\alpha^{2}+\alpha +\beta \alpha -\beta \alpha^{3}+\delta \alpha +3\delta \alpha^{2}-\delta +\delta \alpha^{3}}{{\left(\alpha -1\right)}^{2}}>0, \end{array}$$

and

(16) $$\frac{3\alpha^{3}-4\alpha^{2}+\alpha +\beta \alpha -\beta \alpha^{3}+\delta \alpha +3\delta \alpha^{2}-\delta +\delta \alpha^{3}}{\alpha -1}>-\frac{-3\alpha -2\alpha^{2}+1}{2},$$

for which the equilibrium point $E_{5}$ of the system (8) is of a saddle focus type, and the condition (5) is satisfied. The solution of the system of inequalities (15) and (16) determines the parameter region:

(17) $$0<\alpha <\frac{\sqrt{17}-3}{4},\;\beta >1,\;0<\delta <\frac{7\sqrt{17}+33}{3\sqrt{17}-5}\beta +\frac{61\sqrt{17}-251}{3\sqrt{17}-5},$$

for which the Shilnikov theorem for the system (8) is satisfied. ☐

## 4. Numerical Results

**Figure 1:**
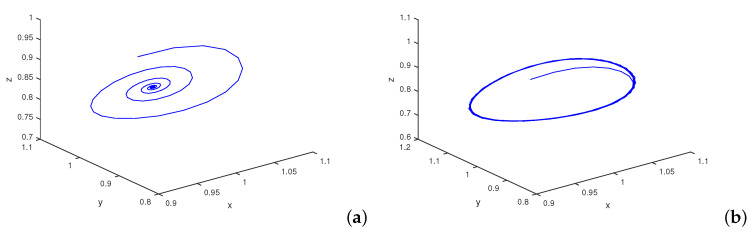

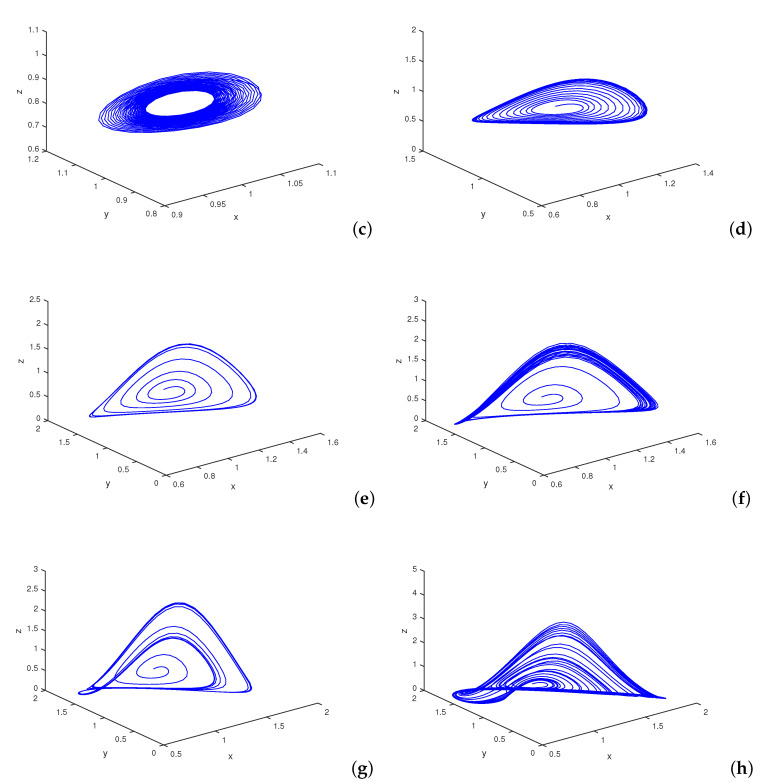
Transition to Shilnikov chaos in the system (7). The values of system parameters are $\eta_{1}=0.6,\;\eta_{2}=0.1,\;\eta_{4}=0.02$. We vary only the value of $\eta_{3}$. Figure (**a**): $\eta_{3}=1$. There is a stable equilibrium state. Figure (**b**): $\eta_{3}=1.391$. The cyclic state after the Hopf bifurcation is purely presented. Figure (**c**): $\eta_{3}=1.4$. The trajectory spirals onto the limit cycle. Figure (**d**): $\eta_{3}=1.5$. The saddle focus $E_{6}$ already has approached. Figure (**e**): $\eta_{3}=1.7$. The unstable manifold of the saddle focus is clearly visible. Figure (**f**): $\eta_{3}=1.9$. Periodic motion is observed. Figure (**g**): $\eta_{3}=2$. Period double cascade is observed. A homoclinic loop appears. Figure (**h**): $\eta_{3}=2.3$. Chaotic motion already exists. With further increasing value of $\eta_{3}$ the attractor again is reduced to a multi–periodic cycle, as at $\eta_{3}=2.5$ the periodic motion vanishes.

The process of the appearance of Shilnikov chaos in the system (8) is illustrated in Figure 1. We use $\eta_{3}$ as a control parameter in order to present the evolution of the attractor. The starting point for the calculated trajectories is (0.95, 0.95, 0.95), which is in the vicinity of the fixed point $E_{8}$. As Figure 1 shows, initially, the system (8) has a stable equilibrium state ($E_{8}$) for $\eta_{3}<1.391$ (Figure 1a). With an increasing value of $\eta_{3}$, the equilibrium point becomes unstable, and by means of a supercritical Hopf bifurcation at $\eta_{3}=1.391$, a limit cycle appears (Figure 1b). Figure 1c,d illustrate the evolution of this limit cycle with an increasing value of $\eta_{3}$. We observe that when $\eta_{3}>1.391$, initially the trajectory spirals onto the limit cycle of increasing size (Figure 1c), whereas at $\eta_{3}>1.5$, this orbit becomes a boundary of the unstable manifold of the saddle focus equilibrium point ($E_{5}$) that spirals onto it (Figure 1d,e). Figure 1f shows the increasing influence of this saddle focus when further increasing the value of $\eta_{3}$. Finally, when $\eta_{3}⪰2$ period doubling cascade is observed, and the mechanism of Shilnikov holds: The unstable 1D manifold of the saddle focus touches its stable 2D manifold, thereby forming the aforementioned homoclinic loop of the saddle-focus (Figure 1g). After that, numerous quasiperiodic orbits around the homoclinic loop occur, as the attracting set already contains a set of complex structures; i.e., a spiral strange attractor has appeared (Figure 1h).

The numerical results show several possible scenarios for evolution of the influence of the studied sectors. Scenario 1 is shown in Figure 1a. In this scenario, the system arrives after some transition time to an equilibrium state where the influences of the three sectors are fixed (fixed point) and the system remains at this equilibrium if the values of the parameters do not change. This scenario can be destabilized by changes to the system parameters, and then another scenario (Scenario 2) can appear: after some transition time wherein the influences of the sectors begin to oscillate, one observes periodic oscillations of the influences of the three sectors, and this is represented as a limit cycle on Figure 1b–f. Such behavior is rarely observed and what is much more probable is scenario 3: chaotic changes of the influences of the three sectors—Figure 1g,h. In this scenario an equilibrium state is destabilized and there is chaotic motion of the values of the quantity characterizing the influence. This chaotic motion is between two unstable equilibrium states described by the fixed points corresponding to the Shilnikov chaos. Scenario 3 can be destabilized by changing the parameters, and the scenario 2 or scenario 1 can become stable again.

## 5. Concluding Remarks

In this paper we show that when the influence of the NGO sector is taken into account, the behavior of a national system may become complicated. We note that the equilibrium point $E_{8}$ describes the situation when the influences of the public and private sector are the same, and the influence of the NGO sector is smaller than each influence of the other two sectors. The saddle focus $E_{5}$ corresponds to situation in which the influence of the NGO sector is 0 and the influence of the private sector is larger than its influence at the point $E_{8}$. The observed chaotic attractor is connected to a situation in which the influence of the private sector increases at the expense of decreasing influence of NGO sector, and then the influence of NGO sector increases and at the same time the influence of the private sector decreases. In this process the influence of the public sector moves between the values of the fixed points $E_{5}$ and $E_{8}$. In other words, the Shilnikov chaos corresponds to a situation in which the competition between the private and NGO sectors leads to large changes in the influence of the NGO sector (this influence can even become very small), whereas the influences of the other two sectors oscillate in irregular ways. Changes to the values of the parameters of the model system can lead to different scenarios. For example, there can be equilibrium between the influences of the three sectors (corresponding to a fixed point), or the values of the quantity corresponding to the influences can oscillate over time (which correspond to the attracting sets which are limit cycles).

## References

[1] Chian A.C.-L. (2007). Complex Systems Approach to Economic Dynamics.

[2] Metcalfe J.F., Foster J. (2004). Evolution and Economic Complexity.

[3] Brian Arthur W. (2015). Complexity and the Economy.

[4] Schulz M. (2003). Statistical Physics and Economics.

[5] Richmond P., Mimkes J., Hutzler S. (2013). Econophysics and Physical Economics.

[6] Mantegna R.N., Stanley H.E. (2004). Introduction to Econophysics.

[7] Zhang W.-B. (2005). Differential Equations, Bifurcations and Chaos in Economics.

[8] Rosser J.B. (2000). From Catastrophe to Chaos: A General Theory of Economic Discontinuities.

[9] Lorenz H.-W. (1989). Nonlinear Dynamical Economics and Chaotic Motion.

[10] Braat L.C., van Lierop W.F.J. (1987). Economic-Ecological Modeling.

[11] Dendrinos D. (1992). The Dynamics of Cities: Ecological Determinism, Dualism and Chaos.

[12] Lotka A.J. (1910). Contribution to the Theory of Periodic Reaction. J. Phys. Chem..

[13] Lotka A.J. (1925). Elements of Physical Biology.

[14] Volterra V. (1931). Lessons on the Mathematical Theory of Struggle for Life (Original: Leçons sur la théorie mathématique de la Lutte pour la vie).

[15] Nijkamp P., Reggiani A. (1998). The Economics of Complex Spatial Systems.

[16] Arbia G. (2006). Spatial Econometrics.

[17] Dimitrova Z.I., Vitanov N.K. (2000). Influence of Adaptation on the Nonlinear Dynamics of a System of Competing Populations. Phys. Lett. A.

[18] Dimitrova Z.I., Vitanov N.K. (2001). Adaptation and its Impact on the Dynamics of a System of Three Competing Populations. Physica A.

[19] Dimitrova Z.I., Vitanov N.K. (2004). Chaotic Pairwise Competition. Theor. Popul. Biol..

[20] Dimitrova Z.I., Vitanov N.K. (2001). Dynamical Consequences of Adaptation of the Growth Rates in a System of Three Competing Populations. J. Phys. A Math. Gen..

[21] Dimitrova Z.I., Vitanov N.K. (2005). Shilnikov Chaos in a Generalized System for Modeling Dynamics of Competing Populations. C. R. L’Acade’Mie Bulg. Des Sci..

[22] Palatella L., Perell J., Montero M., Masoliver J. (2004). Activity Autocorrelation in Financial Markets. Eur. Phys. J. B.

[23] Sonubi A., Arcagni A., Stefani S., Ausloos M. (2016). Effects of Competition and Cooperation Interaction Between Agents on Networks in the Presence of a Market Capacity. Phys. Rev. E.

[24] Sabatelli L., Richmond P. (2004). A Consensus-Based Dynamics for Market Volumes. Physica A.

[25] Richmond P., Sabatelli L. (2004). Langevin Processes, Agent Models and Socio-Economic Systems. Physica A.

[26] Vitanov N.K., Dimitrova Z.I., Ausloos M. (2010). Verhulst–Lotka–Volterra (VLV) Model of Ideological Struggle. Physica A.

[27] Vitanov N.K., Ausloos M., Rotundo G. (2012). Discrete Model of Ideological Struggle Accounting for Migration. Adv. Complex Syst..

[28] Ausloos M., Diricks M. (2006). The Logistic Map and the Route to Chaos.

[29] Vano J.V., Wildenberg J.C., Anderson M.B., Noel J.K., Sprott J.C. (2006). Chaos in Low-Dimensional Lotka–Volterra Models of Competition. Nonlinearity.

[30] Roques L., Chekroun M.D. (2011). Probing Chaos and Biodiversity in a Simple Competition Model. Ecol. Complex..

[31] Wang R., Xiao D. (2010). Bifurcations and Chaotic Dynamics in a 4-dimensional Competitive Lotka–Volterra System. Nonlinear Dyn..

[32] Shilnikov L.P. (1965). A Case of the Existence of a Denumerable Set of Periodic Motions. Sov. Math. Dokl..

[33] Arneodo A., Coullet P., Tresser C. (1980). Occurence of Strange Attractors in Three-Dimensional Volterra Equations. Phys. Lett. A.

[34] Afraimovich V.S., Gonchenko S.V., Lerman L.M., Shilnikov A.L., Turaev D.V. (2014). Scientific heritage of L.P. Shilnikov. Regul. Chaotic Dyn..

